# Assessment of volumetric absorbed dose for mobile fluoroscopic 3D image acquisition

**DOI:** 10.1002/acm2.12108

**Published:** 2017-06-06

**Authors:** Stephanie Leon

**Affiliations:** ^1^ Department of Radiology University of Florida Gainesville FL USA

**Keywords:** 3D fluoroscopy, cone beam CT, CTDI, mobile c‐arm, volumetric dose

## Abstract

Mobile fluoroscopy (c‐arm) units offering 3D image reconstruction are becoming more common in surgical settings. Although these images are “CT‐like” and sometimes replace the postoperative CT, the acquisition is technically very different from a traditional CT acquisition. Dose assessment is complicated by a large beam width, automatic exposure rate control, and a rotation of less than 360°. The purpose of this work was to explore the impact of these factors on the volumetric dose calculation and to provide practical recommendations for clinical physicists assessing dose from these units using commonly available equipment. CTDI_W_ was calculated using the IAEA method for dosimetry of wide beams and compared to scans of the 32‐cm CTDI phantom using the full beam width and a 20‐mm collimated beam width. The impact of the partial rotation on the CTDI_W_ calculation was assessed by acquiring measurements at four and twelve positions on the phantom periphery. For the system tested, the CTDI_W_ was calculated to be 16.1 mGy using the IAEA method with default clinical protocol. Results showed that measuring CTDI_W_ with the full beam width or a collimated beam width alone resulted in CTDI values of 19.0 mGy and 19.5 mGy, respectively. Using four peripheral measurements instead of 12 resulted in a difference of 4% for a collimated beam and 6% for an open beam. Variations in positioning on the order of a few centimeters resulted in a variation of only 4% with an open beam. The excellent reproducibility of the measurements using the full beam width suggests that this simple method is adequate for year‐to‐year comparisons. In contrast, the IAEA method is difficult to employ, particularly with 180° acquisitions. Use of peripheral measurements in excess of the usual four is time‐consuming and not necessary for most applications obtained with the geometry specific to this system.

## 1 INTRODUCTION

**Figure 1:**
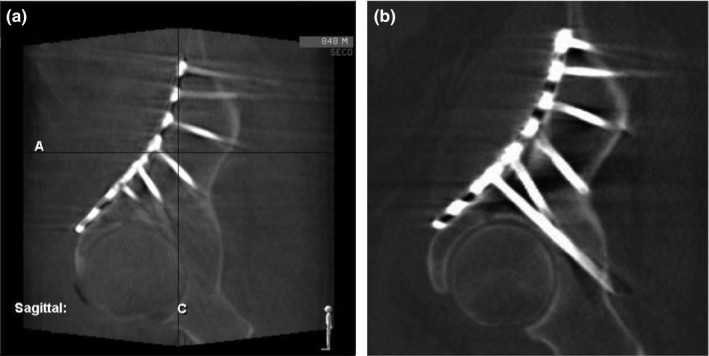
Postsurgical sagittal reconstruction of patient hardware from the (a) Ziehm c‐arm and (b) multislice CT scanner.

Mobile fluoroscopy (c‐arm) units are commonly used during surgical procedures. Some units now possess the ability to perform rotating acquisitions with 3D image reconstruction to produce images similar to traditional CT images (Fig. 1), and are increasingly being used to replace the postoperative CT. However, the unit investigated in this study, the Ziehm Imaging (Orlando, FL, USA) Vision RFD 3D mobile c‐arm, does not provide any volumetric dose metrics. The only “dose” quantities provided are the beam‐on time and dose to a reference point used for fluoroscopic imaging. The typical volumetric dose from the 3D acquisition is unknown.

Dose assessment using the CTDI_W_ is complicated due to the way in which the images are acquired. The beam width is approximately 150 mm at isocenter^1^ and cannot be collimated during the acquisition. The unit uses automatic exposure rate control (AERC) during the acquisition, which can result in the mA changing throughout the acquisition as the patient thickness changes due to changing projection angle. This AERC adjusts the mA “on the fly” during the rotation as a function of radiation intensity incident on the detector, like that of a standard fluoroscopy unit. Additionally, the system uses a 180° rather than a 360° scan, and the data are acquired with a complicated tube motion consisting of two linear 7.5° translations and a 165° rotation.^2^ The geometry of the system and a detailed description of the tube motion are well‐described in other publications.^1^, ^3^

CTDI_W_ is traditionally calculated using the weighted ratio of dose measurements made at the center and periphery of a standardized acrylic phantom, as follows:

(1) $${\mathrm{CTDI}}_{W}=\left[2/3\times {\mathrm{CTDI}}_{100(\mathrm{periphery})}+1/3\times {\mathrm{CTDI}}_{100(\mathrm{center})}\right]$$

where CTDI_100_ is the measurement acquired with a 100‐mm‐long pencil ion chamber. The value used for the periphery is either the average of measurements acquired at the four cardinal positions (3 o'clock, 6 o'clock, 9 o'clock, and 12 o'clock), or at the 12 o'clock position alone. For a rotation of less than 360°, however, it is questionable whether four positions are sufficient, since either one or two of the measurement points may be outside the beam depending on the relative positions of the x‐ray tube arc and dosimeter. Previous studies have employed the use of four^4^, ^5^ and eight measurement points,^6^, ^7^, ^8^ but they did not compare the accuracy of differing numbers of points. A study using a fixed c‐arm with 200° rotation found a variation of up to 10% in the CTDI_W_ calculated when measurements were acquired at the four cardinal positions and when they were rotated by 45°.^7^

The use of the 100‐mm pencil chamber was developed for narrow‐beam CT scanners, but a number of modern CT units now have beam widths that exceed 100 mm. The traditional pencil chamber is insufficient to measure the entire beam in these scanners. Although alternate measurement techniques have been proposed to better measure wide beams,^6^, ^8^, ^9^, ^10^, ^11^, ^12^, ^13^, ^14^, ^15^, ^16^ at this time, most clinical physicists do not have the equipment necessary to follow these recommendations. The International Atomic Energy Agency (IAEA) released a report in 2011 detailing a technique to calculate a CTDI for wide beam scanners using the standard 100‐mm pencil chamber and 32‐cm acrylic phantom,^16^ which was based on recommendations from the International Electrotechnical Commission^17^ and recommended for use by the wide‐beam CT dosimetry working party of the Institute of Physics and Engineering in Medicine.^18^ This methodology is used in this study. The technique involves making measurements both in the acrylic phantom and free‐in‐air and calculating the CTDI_100_ as follows:

(2) $$CTDI_{100,N\times T}=CTDI_{100,ref}\times \frac{CTDI_{free-in-air,N\times T}}{CTDI_{free-in-air,ref}}$$

where $CTDI_{100,N\times T}$ is the calculated CTDI with the full beam width in the acrylic phantom, $CTDI_{100,ref}$ is acquired with a collimated beam of 20 mm (or as close as possible) in the acrylic phantom, $CTDI_{free-in-air,ref}$ is acquired with the collimated beam width with the chamber suspended free‐in‐air at isocenter, and $CTDI_{free-in-air,N\times T}$ is acquired free‐in‐air with the full beam width. For this last measurement, the chamber is translated in incremental steps over multiple acquisitions to fully cover the beam, and the measurements are integrated. Further details regarding this method can be found in the IAEA publication cited. The calculated $CTDI_{100,N\times T}$ values can then be used to calculate the CTDI_W_ using the standard method.

This study has several goals: to determine the CTDI_W_ from the 3D acquisition; to determine whether four peripheral measurements are sufficient to obtain a stable average; and to see whether scanning the acrylic phantom with the full beam width can be employed as a simpler method for clinical physicists to use for routine measurements than the IAEA method.

## 2 METHODS

**Figure 2:**
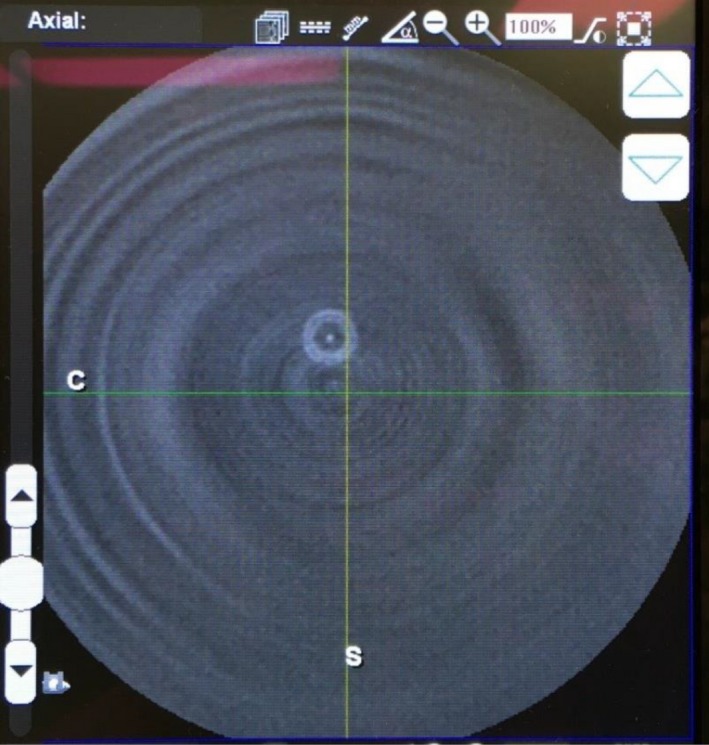
The centering of the phantom or chamber at isocenter was verified from the reconstructed image. This image demonstrates a slight misalignment of the pencil chamber, indicated by the location of the chamber cross‐section superior to the isocenter crosshairs. This image was acquired with the chamber free‐in‐air, but it is equally visible in the phantom.

The system tested for this study was a Ziehm Vision RFD 3D mobile c‐arm. To simulate clinical operating conditions, the operating table used clinically (Mizuhosi OSI 5803 Advanced Control Base with Tempur‐pedic pad) was used for all measurements except those acquired free‐in‐air. The 32‐cm CTDI phantom was positioned on the table pad and centered from left‐to‐right. The c‐arm was positioned so that the phantom was aligned correctly for the c‐arm rotation using the alignment lasers. Alignment was verified on the reconstructed 3D image (Fig. 2).

The clinical protocol for the sacrum was selected for this study, since that is a commonly used protocol at our institution; however, the anatomical selection did not appear to affect the acquisition techniques. The default acquisition technique used clinically employs a tube current of 100 kV, a pulse rate of eight pulses per second and a pulse width setting of 58% (which corresponds to a pulse width of 23 ms^19^). The default technique was not altered for this study. The Ziehm c‐arm utilizes AERC during the acquisition, and it cannot be disabled. The acquisition starts with a linear translation lasting approximately 7 s, which always occurs at 15 mA. The mA then adjusts to the level appropriate for the object for a rotation lasting approximately 37 s. The final linear translation lasts approximately 3 s and occurs at the final mA used during the rotation. These motions are managed by the c‐arm robotics and are not controlled by the user. When centered on the table with the acrylic phantom in place, the phantom alone was sufficient to drive the tube current to the maximum available on the unit, 80 mA, for the entire scan after the initial translation. Thus, the complication of a varying tube current during the phantom data acquisition was avoided.

Each individual image acquisition requires several minutes due to the long acquisition time, required prescan collision check, and automated c‐arm motions. To speed the measurement process, up to three dosimeters and pencil ion chambers were used during each image acquisition so that multiple measurements could be acquired simultaneously: an MDH model 1015, a RadCal model 9015, and a RaySafe model X2. Prior to making measurements with the Ziehm c‐arm, a traditional CT scanner (Siemens Somatom Definition AS) was used to assess the agreement and reproducibility of measurements made with the differing dosimetry systems. Scans were acquired with a manual technique and 38.4‐mm collimation width using a single axial rotation at 100 kV. Helical scans at 120 kV covering the entire phantom width were also performed, since the Ziehm c‐arm uses a cone beam capable of irradiating the entire phantom. Each measurement was acquired three times. Each meter demonstrated a standard deviation of less than 1% among repeat measurements, for all scan conditions. Calculations of CTDI_vol_ found using each meter all agreed to within 1% of each other for the axial and helical scans.

### 2.A Assessment of CTDI_W_

**Figure 3:**
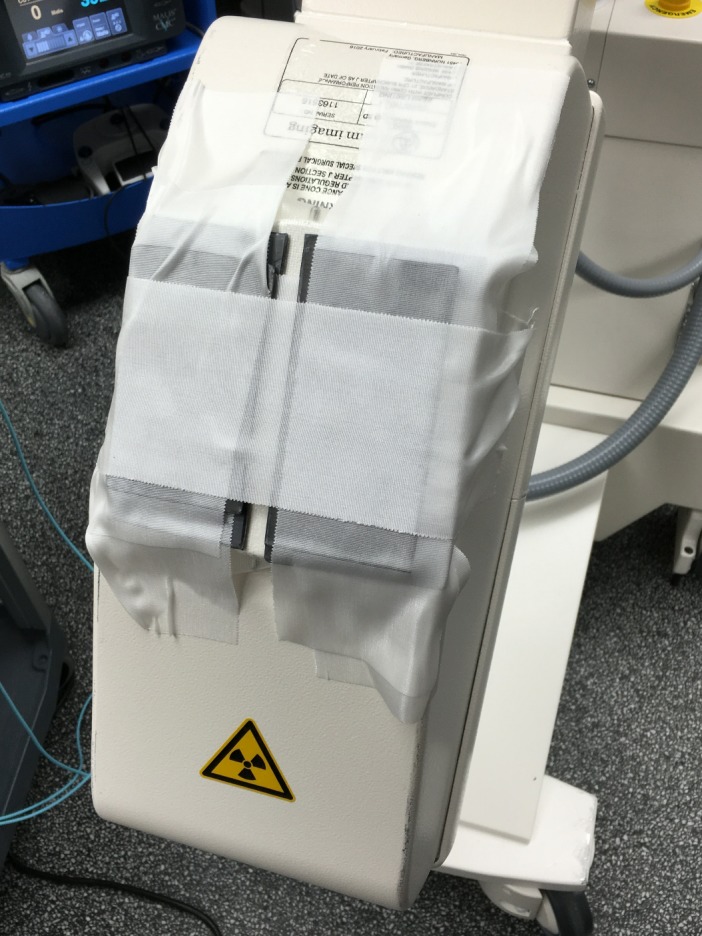
Photograph of the lead pieces used to collimate the x‐ray beam.

**Figure 4:**
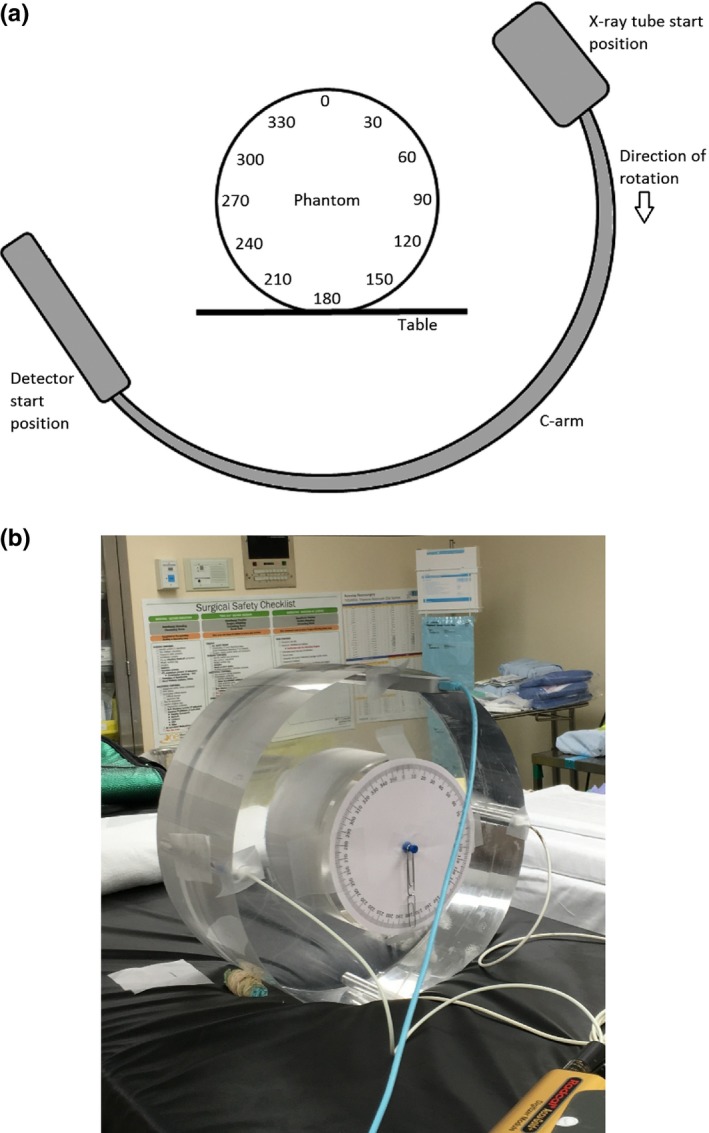
The experimental set‐up, showing (a) a diagram of the positions at which the measurements were acquired and (b) a photograph of the phantom with ion chambers and angle indicator in place.

For this assessment, the IAEA method was used to calculate the CTDI_W_. The IAEA method requires collimation of the beam to a width as close as possible, but not more than, 20 mm. The Ziehm c‐arm does not allow collimation during the 3D acquisition, so lead pieces were taped to the exit of the tube housing to collimate to a beam width of 19.6 mm at isocenter. The lead pieces were approximately 3‐mm thick and of an appropriate size to cover the tube window. Based on the system geometry, the distance between the lead pieces required to achieve the required beam width was 8 mm at the tube housing (Fig. 3). The lead pieces were carefully adjusted to ensure an 8‐mm gap across the entire length, and were not moved for the duration of the measurements. Because the tube current was already at the maximum available, the addition of the collimator did not affect the mA for the phantom measurements. Exposure measurements were acquired at the center position and at 12 peripheral positions evenly spaced in increments of 30°. The 12 o'clock position was considered “zero degrees,” with the angle increasing in the clockwise direction toward the x‐ray tube start position [Fig. 4(a)]. The 30‐degree increments were obtained simply by rotating the phantom to relocate the peripheral holes; the angles were measured by affixing a simple paper circle with the angles denoted to the phantom and indication of the current angle with a pendulum [Fig. 4(b)]. Reproducibility was also checked for seven measurements at the center and three measurements at each of the cardinal positions (0, 90, 180, and 270°). The coefficients of variation at each measurement position ranged from 0.05% at 270° to 5% at 0°. The coefficient of variation was equal to 0.3% for the values of CTDI_W_ calculated using those five measurements.

After acquiring the measurements in the phantom, the free‐in‐air measurements were obtained following the IAEA instructions. The detector was covered with a lead apron to increase the mA and reduce the risk of ghosting. Only one apron was used due to the difficulty of securely taping it to the detector during the rotation and the need to remove it during each preacquisition “collision check.” Because the lead apron was not sufficient to drive the AERC to maximum, reproducibility was assessed again with three measurements, with a coefficient of variation of less than 1%. The mA displayed by the system was identical for all three measurements. For the free‐in‐air measurement with the uncollimated beam, the ion chamber was translated twice (for a total of three acquisitions) to provide complete coverage of the beam without overlapping.

For the collimated free‐in‐air measurement, the tube current was 28.7 mA (after the initial translation at 15 mA). For the uncollimated free‐in‐air measurement, it was 12.4 mA for all three acquisitions. For the purpose of the $CTDI_{100,N\times T}$ calculation, the air kerma measurements were scaled to a common mAs. The amount of time spent at each mA value was taken into account, as was the portion of the linear translations during which the ion chamber would not have been in the x‐ray beam.

### 2.B Comparison to phantom‐only measurements

The IAEA method involves three sets of measurements with differing setups. For routine testing, it would be ideal to simplify this testing scheme. The CTDI_W_ calculated from the IAEA method (CTDI_W‐I_) was compared to a CTDI_W_ calculated from the collimated phantom measurements alone (CTDI_W‐c_) and from measurements with the uncollimated (open) beam (CTDI_W‐o_). For calculation of CTDI_W‐c_, the same measurements acquired using the 20‐mm collimated beam in the phantom for the IAEA method were used to calculate a CTDI_W_ using Eq. (1). For the open beam, the measurements were repeated without collimation. For both scenarios, the measurements were acquired at all 12 angles described previously.

To gauge the impact of small variations in phantom positioning, the measurements for calculating CTDI_W‐o_ were conducted a total of three times at the cardinal measurement positions, with the phantom removed from the table and the c‐arm repositioned between trials. Although a reasonable attempt was made to position everything accurately using the alignment lasers, less exacting attention was paid to perfect positioning during these trials. Because the purpose of this study is to determine factors important to the clinical physicist who may be testing such a unit, the effect of phantom positioning is an important factor to consider when comparing year‐to‐year results from annual testing.

### 2.C Number of measurements required

For the purpose of routine evaluation, it is preferable to limit the number of measurements that need to be acquired. The data from the four cardinal angles acquired under each condition were used to calculate the CTDI_W_ for comparison to the 12‐angle scenario. This test was done both for the collimated and uncollimated measurements.

## 3 RESULTS AND DISCUSSION

**Figure 5:**
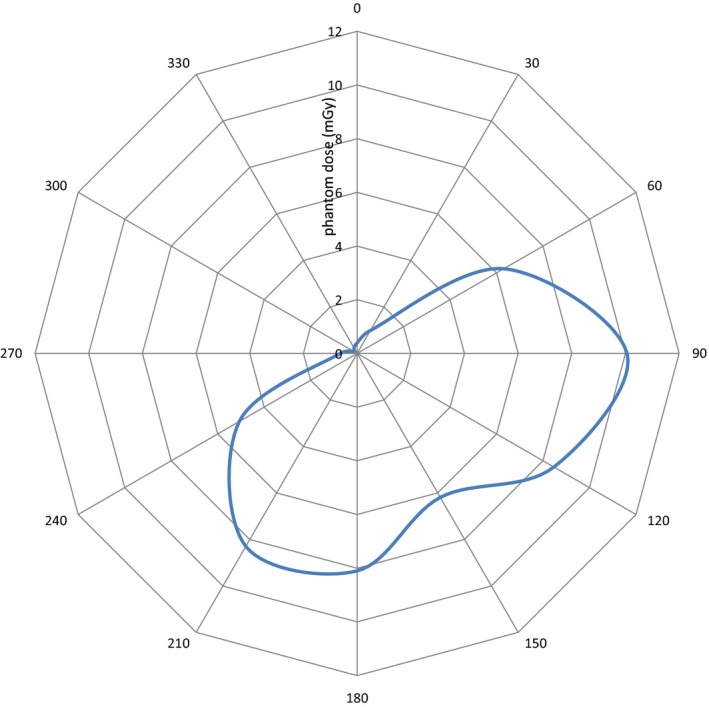
Phantom dose measurements (mGy) as a function of angle in the phantom, using a 20‐mm collimated beam width.

Using the IAEA method, the CTDI_W‐I_ was calculated to be 16.1 mGy (Table 1). Caution must be taken when comparing this value to the CTDI_vol_ reported for a conventional CT unit. (For the case of the c‐arm, CTDI_W_ and CTDI_vol_ are equivalent, since there is no table movement.) For both types of units, the CTDI_vol_ provides an estimate of the energy imparted to a standardized phantom, averaged over the scan volume. It can be used as an index to compare systems and track performance (the purpose of CTDI). However, the air kerma measurements acquired in the phantom (Fig. 5) clearly show the asymmetric dose distribution resulting from the 180‐degree rotation. Due to this asymmetry, it is not appropriate to use the CTDI_W_ to calculate an effective dose using the k‐factor method^20^ commonly used for standard CT examinations because some organs within the scan volume will be exposed to far more radiation than others. In this study, measurements among the 12 different angles varied by over a factor of 50 (maximum at 90° and minimum at 300°), as compared to about a factor of 2 commonly seen in conventional CT. The specific way in which the dose distribution overlays the organs, and the radiosensitivity of these organs, must be considered if one is to estimate an effective dose. To further complicate matters, many intraoperative clinical images are not acquired with the patient centered at isocenter. The calculated CTDI_W_ therefore cannot be related to stochastic risk without extensive modeling and calculation.

**Table 1:** CTDI_W_ measurements made, with 95% confidence intervals. The number of positions indicated refers to the number of measurement points at the phantom periphery: either 4 (spaced every 90°) or 12 (spaced every 30°). Trials 1–3 with the open beam involved repositioning the phantom and c‐arm between trials, to assess the impact of small differences in positioning

Measurement method	CTDIW (mGy)
IAEA method	16.1 (10.6, 21.6)
Open beam in phantom (12 positions)	19.0 (17.3, 20.8)
Collimated beam in phantom (12 positions)	19.5 (17.9, 21.0)
Open beam trial #1 (4 positions)	21.7 (19.9, 23.4)
Open beam trial #2 (4 positions)	21.5 (19.8, 23.3)
Open beam trial #3 (4 positions)	20.1 (18.4, 21.8)
12 positions (collimated)	19.5 (17.9, 21.0)
4 positions (collimated)	20.2 (18.7, 21.8)
12 positions (open)	19.0 (17.3, 20.8)
4 positions (open)	20.1 (18.4, 21.8)

Using measurements acquired in the phantom alone to generate a CTDI_W_ resulted in CTDI_W‐c_ = 19.5 mGy (21% higher than CTDI_W‐I_) and CTDI_W‐o_ = 19.0 mGy (18% higher than CTDI_W‐I_). Of interest to the clinical physicist is that the value for CTDI_W‐o_ showed little change among the trials in which both the phantom and c‐arm were repositioned, displaying a variation of only 4%; this suggests that year‐to‐year reproducibility of this method should be adequate for routine surveys. There was also very little difference in the calculated CTDI_W_ when phantom measurements were made with and without collimation. The time‐consuming addition of collimation is therefore unnecessary for routine testing.

Three publications assessing the dose to phantoms from c‐arm systems with half‐rotation orbits were found for comparison to the values generated in this study (however, all used a Farmer ionization chamber rather than 100‐mm pencil chamber). A study by Fahrig et al. that measured the dose from a fixed c‐arm found a dose of 39.6 mGy in a 16‐cm phantom at 109 kV.^6^ A second study by Schafer et al. found that the dose to a 32‐cm phantom from a prototype mobile c‐arm was in the range of 3.70–12.50 mGy, depending on the protocol.^21^ An earlier study by Daly et al. measuring the dose from the same prototype c‐arm system as Schafer et al. reported only the dose at isocenter of a 16‐cm phantom, but they found that doses of 3–10 mGy using a 100 kV beam provided adequate image quality depending on the imaging task.^22^ None of these studies used the Ziehm system, but comparison to these results indicates that the Ziehm mobile c‐arm performs within the range of dose levels observed from other comparable systems.

Only one other study comparing the IAEA method to phantom‐only measurements was found: Gancheva et al. used a traditional (360° scan) CT unit having a 16‐cm beam width and found that CTDI_W‐c_ was 11.3% higher than CTDI_W‐I_ and CTDI_W‐o_ was 41% lower than CTDI_W‐I._ 23 It is initially surprising that CTDI_W‐o_ was found to be higher than CTDI_W‐I_ in the current study, as numerous studies have indicated that CTDI_W‐o_ for a wide beam is expected to be artificially low when using a 100‐mm ion chamber and 150‐mm acrylic phantom.^5^, ^7^, ^9^, ^10^, ^11^, ^12^, ^14^, ^15^, ^16^, ^23^ Rather than contradicting the existing literature, this is most likely an indication that the IAEA method is not ideal for use with 180° acquisitions. The study by Gancheva et al. used a 360° acquisition; the other studies finding that CTDI_W‐o_ tends to underestimate the “true” CTDI_W_ compared it to measurements or simulations of longer phantoms, not to the IAEA method. The potential difficulty with the IAEA method is the in‐air measurement at isocenter. The dose distribution shown in Fig. 5 demonstrates that the dose drops quickly in the regions not directly covered by the arc of rotation. The chamber at isocenter would be positioned at the edge of this region, making the measurements susceptible to small inaccuracies in positioning. To test this idea, an additional in‐air measurement at isocenter was made during a separate measurement session. Despite careful positioning with the lasers, this new exposure measurement and the original measurement differed by 12% (this error is reflected in the 95% confidence interval shown in Table 1). These effects are not noted with the phantom measurements due to scatter and the weighted averaging with the peripheral measurements that takes place in the CTDI_W_ calculation.

The open beam method is by far the simplest method to employ, since it does not require the creation of an external collimator nor the use of in‐air measurements. If the sole purpose of the measurement is to track year‐to‐year performance on the same unit, the difference from the IAEA method is not of much importance, and the open beam method should suffice. However, if the goal is to compare different systems, which may have different fields of view, then use of a standardized method to account for beam thickness is required. The IAEA method is not recommended for this purpose if the system employs a rotation of less than 360°. Accurate measurements would likely require specialized equipment such as extended phantoms, longer pencil chambers, or small ion chambers, as recommended by other researchers.^5^, ^6^, ^9^, ^10^, ^11^, ^12^, ^13^, ^14^, ^23^

Surprisingly, the CTDI_W_ did not change much when calculated with only four peripheral measurements instead of 12. With the collimated beam, the calculated CTDI_W_ increased by 4% when using the four cardinal measurements instead of all 12. With the open beam, the calculated CTDI_W_ increased by 6% using four measurements instead of 12. However, caution should be exercised in applying these data to systems which may have differing rotation arcs or that start and stop at different angles with respect to the measurement positions.

## 4 CONCLUSIONS

The purpose of this study was to assess the practical aspects of measuring a volumetric dose from a mobile c‐arm with 3D image acquisition. Methodology was restricted to the use of equipment commonly available to the clinical physicist: the 32‐cm acrylic CTDI phantom, 100‐mm pencil ion chamber, and small lead sheets. CTDI_W_ was calculated using the IAEA method for wide beams and found to be 16.1 mGy. Comparison was made to CTDI_W_ calculated using just phantom measurements, with the hope that a simpler method would suffice. When measurements were made in the phantom with an open beam, they resulted in a CTDI_W_ of 19.0 mGy, 18% greater than with the IAEA method. Measurements made in the phantom with a collimated beam resulted in a CTDI_W_ of 19.5 mGy. The discrepancy between the phantom‐only measurements and the IAEA method may be due to the difficulty of employing the IAEA method with a 180° acquisition. However, reproducibility using the open beam method was excellent, so use of this simpler method for the sole purpose of tracking performance year‐to‐year should be acceptable. The use of added collimation produces CTDI_W_ values very similar to the open beam, suggesting that collimation is unnecessary for routine testing. The use of greater than four peripheral measurements in the phantom is time‐consuming and unnecessary with the geometry of this unit.

## ACKNOWLEDGMENTS

The author would like to thank Justin B. Faul, RT, and Joshua L. Gary, MD. Their assistance with this project was invaluable. The author is also thankful for the use of clinical imaging equipment at Memorial Hermann Hospital – Texas Medical Center in Houston, TX. Publication of this article was funded in part by the University of Florida Open Access Publishing Fund.

## CONFLICTS OF INTEREST

The author has no conflicts of interest to disclose.
